# Structural Mechanism of Ionic Conductivity of the TRPV1 Channel

**DOI:** 10.1134/S1607672922600245

**Published:** 2023-01-18

**Authors:** Yu. A. Trofimov, A. S. Minakov, N. A. Krylov, R. G. Efremov

**Affiliations:** 1 Shemyakin and Ovchinnikov Institute of Bioorganic Chemistry of the Russian Academy of Sciences, Moscow, Russia; 2 National Research Nuclear University MEPhI (Moscow Engineering Physics Institute), Moscow, Russia; 3 Moscow State University, Moscow, Russia; 4 National Research University Higher School of Economics, Moscow, Russia; 5 Moscow Institute of Physics and Technology, Dolgoprudny, Russia

**Keywords:** TRPV1, ion channels, hydrophobic gate, ionic conductivity

## Abstract

The so-called “hydrophobic gating” is widely discussed as a putative mechanism to control water and ion conduction via ion channels. This effect can occur in narrow areas of the channels pore lined by non-polar residues. In the closed state of the channel, such regions may spontaneously transit to a dehydrated state to block water and ions transport without full pore occlusion. In the open state, the hydrophobic gate is wide enough to provide sustainable hydration and conduction. Apparently, the transport through the open hydrophobic gate may by facilitated by some polar residues that assist polar/charged substances to overcome the energy barrier created by nonpolar environment. In this work, we investigated the behavior of Na^+^ ions and their hydration shells in the open pore of the rat TRPV1 ion channel by molecular dynamics simulations. We show that polar protein groups coordinate water molecules in such a way as to restore the hydration shell of ions in the hydrophobic gate that ensures ion transport through the gate in a fully hydrated state.

In many types of ion channels, certain internal surface areas of their transmembrane pores are lined with non-polar residues forming a kind of “belts” of hydrophobicity. When a channel resides in a closed/inactivated state, this prevents spontaneous diffusion of water and ions through the membrane. At the same time, when a channel is activated and, consequently, the pore radius increases, especially in the narrowest places (the so-called “activation gates”), effective transport of water molecules and ions across the membrane is observed. Such effect is known as “hydrophobic gating,” and it can block water and ion transport without full occlusion of the pore (typically with a radius of C_α_ atoms ≤ 4 Å) [1]. Apparently, the transport through the hydrophobic gates may by facilitated by some polar “helper groups” of amino acid residues that assist polar/charged substances (like water and ions) to overcome the energy barrier created by non-polar environment.

A striking example of a channel with a similar “compensatory mechanism” of operation of a hydrophobic gate is the TRPV1 ion channel. Previously, we performed molecular dynamics (MD) simulations of TRPV1 to study the hydrophobic organization of its pore in open and closed states, and evaluate abnormal dynamics of water molecules in a confined volume of the channel pore [2, 3]. Because the aforementioned problem of ion conductivity through the hydrophobic regions of the pore is still under debate, in this work, we investigated by MD modeling the behavior of Na^+^ ions and the organization of their hydration shells in the open pore of the rat TRPV1 [4], primarily in the vicinity of the activation gate. We show that the structure of the gates is organized in such a way as to restore the ion’s hydration shell. Base on MD results, we propose a mechanism of ion transport, in which the ions pass through the hydrophobic gate in a fully hydrated state due to the coordination of the shell water by polar groups of asparagine residue located in the gate region of the pore.

TRPV1 (Transient Receptor Potential Vanilloid 1) is a nonselective cation channel expressed mainly in sensory neurons, sensitive to high temperature (>43°C), low pH (5.9), mechanical stress and a number of chemical agents such as capsaicin and anandamide [5–7]. TRPV1 is a homotetramer, in the center of which there is a pore formed by transmembrane α-helices of the adjacent monomers. The pore represents a nanometer-scale channel accessible to water molecules and ions, the walls of which have heterogeneous physicochemical properties [2]. There are two “bottle necks” in the pore: the filter and the activation gate. The narrowest region of the filter is polar and composed of the carbonyl atoms of Gly643. The gate of the pore is formed by nonpolar side chains of Ile679 residues, it controls the channel conductivity creating a hydrophobic gate (Fig. 1). The experimental data suggest that the double substitution of Ile679Ala + Ala680Gly seems to make an “always open” phenotype of the channel [8] The polar side chains of Asn676 are oriented inside the pore and their carboxyl groups are directed into the vicinity of the gate—this can prevent the formation of a hydrophobic gate when the channel is active. Asn676 is assumed to be a promoter of the pore hydration [3, 9], and the point mutation of Asn676Ala provides a non-functional phenotype of the rat TRPV1 [10].

**Fig. 1.**
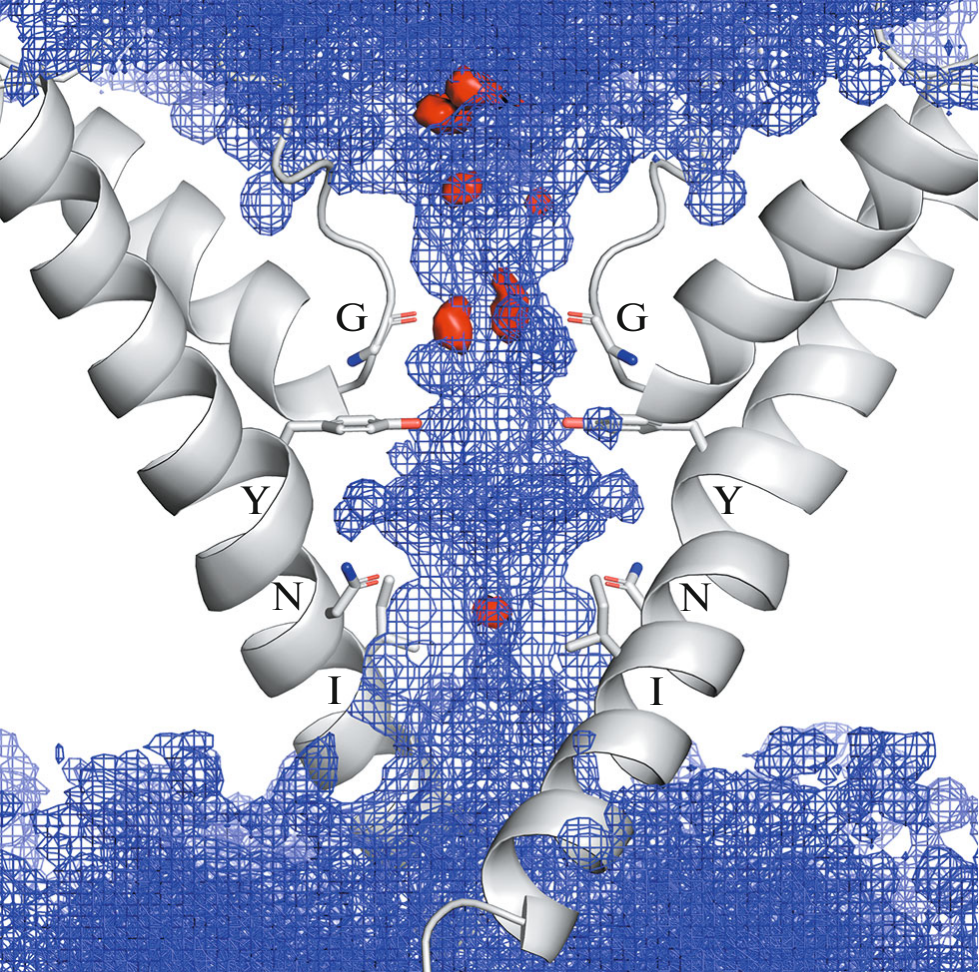
The structure of the TRPV1 pore (PDB ID 7L2W, [4]), only two diagonally arranged subunits are shown. Pore forming S6 and Pore helices are given in a ribbon presentation, pore lining residues Gly643 (G), Tyr671 (Y), Asn676 (N) and Ile679 (I) are shown as sticks and marked. Density distributions of water molecules (blue grid, threshold 0.03 mol/Å^3^) and ions (red surface, threshold 0.03 ion/Å^3^), averaged along the MD are imposed on the pore structure.

In this work, MD modeling of an open TRPV1 channel (PDB ID 7L2W, [4]) embedded into a hydrated lipid bilayer was carried out. Analysis of MD data showed that despite the hydrophobic properties of its walls, the pore is completely filled with water molecules. Na^+^ ions penetrate and localize in the polar filter and in the hydrophobic gate (Fig. 1).

For a detailed analysis of the behavior of Na^+^ ions in the pore, two-dimensional ion density distributions, as well as the average values of Na^+^ coordination number – the number of water and protein oxygens in the first coordination sphere of the ion (CNw and CNp, respectively) were calculated depending on the coordinates along the pore axis (*Z*) and the distance from the axis (Fig. 2). Two maxima are observed in the ion density distribution: at *Z* = 9–13 Å (filter) and at *Z* = –5–0 Å (gate). Localization of ions in the filter is shifted from the pore axis, while two water molecules from the ion coordination sphere are replaced by two oxygen atoms of Gly643, that completes the solvation shell of the ion consisting of 6 oxygen atoms. Following towards the gate, the ions again deviate from the pore axis by 2–4 Å and bind to the oxygen atom of the Asn676 side chain (the lilac region at *Z* = –2.5–+5 Å in Fig. 2с), while losing 2 water molecules. Then the ions enter the gate, restoring their hydration shell to 6 water molecules. In the gate region, the highest ion density was observed on the pore axis, that is, exactly in the “mouth” of the gate.

**Fig. 2.**
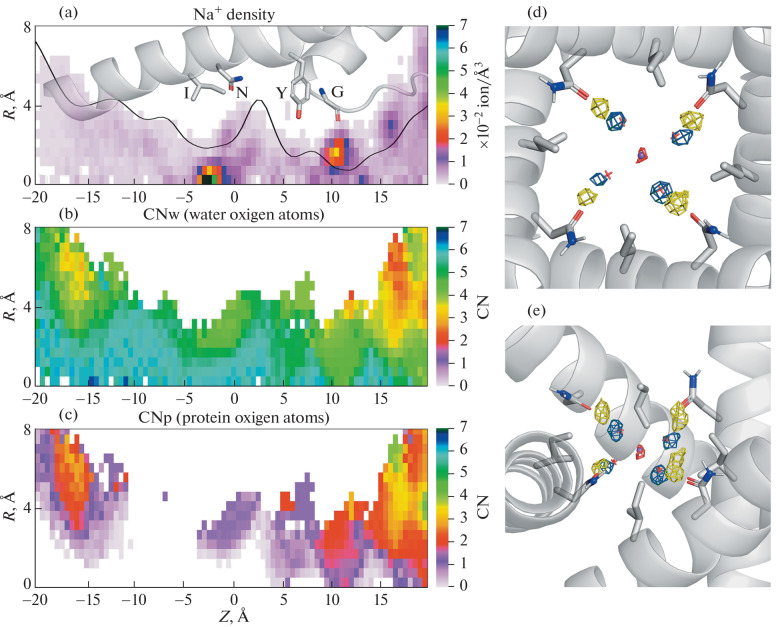
(a) Distribution of Na^+^ density along the pore axis (*Z*) depending on the distance from the axis (*R*). Color scale—density values, ×10^–2^ ion/Å^3^. The location of one of the pore-forming helix S6 is shown. The key residues are indicated: Ile679, Asn676, Tyr671, Gly643. The black line is the pore radius calculated through the volume available to solvent. (b–c) Distributions of CNw and CNp values along the pore axis depending on the distance from the axis, color scale—CN values. (d–e) Distributions of Na^+^ (red), oxygen atoms of water in its hydration shell (blue mash) and water—Asn676 hydrogen bonds (yellow) averaged over MD trajectory. The side chains Asn676 and Ile679 are shown, the purple sphere and red crosses represent Na^+^ and its solvation shell in bulk water: (d) top view; (e) side view.

Spatial distribution of the water density in hydration shell of ions localized in the gate shows that the water molecules are clustered around the ion at the vertices of the square – the base of the octahedron, which corresponds to the shape of the Na^+^ hydration shell in bulk water [11]. The average distance between the oxygens of water and Na^+^ is 2.33 Å, which is close to the bulk value. These water molecules are localized in a hydrophobic environment between the side chains of Ile679, forming hydrogen bonds with oxygens of the side chains of Asn676 oriented to the gate (Figs. 2d, 2e).

Based on the simulation results, we proposed a possible mechanism of Na^+^ transport through the hydrophobic gate of the open TRPV1. In the inner volume of the pore, ions bind to the oxygen atom of the Asn676 side chain of one of the protein subunits, while losing 1–2 water molecules from their hydration shell (Fig. 3b), and then penetrate into the hydrophobic “mouth,” restoring the full hydration shell—6 water molecules (Fig. 3c). The gate structure is organized in such a way that the oxygen atoms of the Asn676 side chains coordinate 4 water molecules, forming hydrogen bonds with them, directly in the gate, in the hydrophobic environment of the Ile679. In this case, the arrangement of waters corresponds to the geometry of the Na^+^ hydration shell in bulk water.

**Fig. 3.**
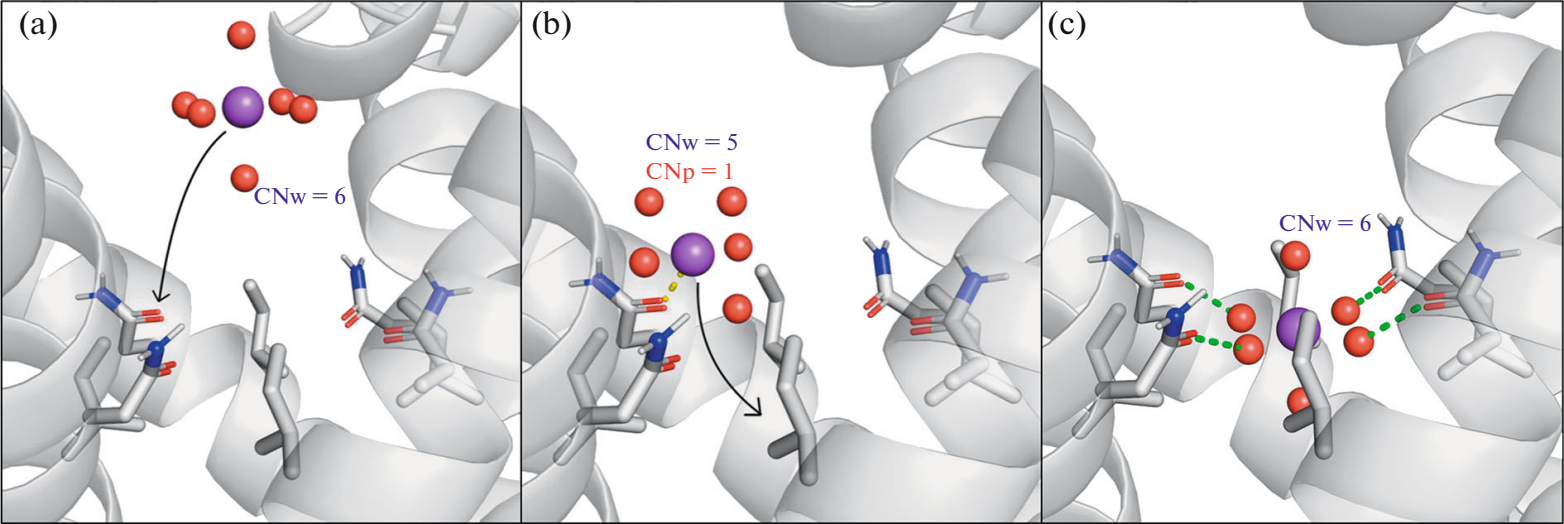
Mechanism of Na^+^ transport through the hydrophobic gate of the open TRPV1. Residues Ile679 and Asn676 are shown. The purple sphere is the Na^+^ ion, the red balls are the oxygen atoms of water molecules, the text shows CN for water (CNw) and protein (CNp). The arrows represent the direction of ion movement through the gate. The yellow dotted line is the contact between the Na^+^ ion and the oxygen atom of Asn676, the green ones are the hydrogen bonds between the oxygen atom Asn676 and the water molecules of the ion hydration shell.

Previously, the presence of hydrophobic gates in ion channels of various types was widely discussed in the literature [1, 12–14]. However, as we know, the only putative mechanism for controlling the conductivity of such gates was an increase in the diameter of a hydrophobic aperture, leading to their hydration and a decrease in the energy barrier for the passage of ions in the hydrated state. Here, using the example of the TRPV1 channel in the open state, it is shown that the geometry of the hydrophobic gate and the distribution of charges in it can be arranged in such a way as to localize waters in an optimal way for ion hydration. According to our MD results, this process in TRPV1 is mainly regulated by the Asn676 residues, which temporally “substitute” 4 water molecules to the Na^+^ hydration shell. TRPV1 is a non-selective cation channel, and another types of ions with various hydration shells can pass through the pore (e.g., square antiprism for K^+^ and Ca^2+^ [11]). In seems probable that flexible Asn676 chains can adopt the coordinated water for different cations, thus ensuring their transport through the gate. The proposed molecular mechanism of cations passage through the hydrophobic gate of the channel has not been formulated before. The results obtained will be useful for understanding the details of the ionic conductivity of both proteins of the TRPV subfamily and other ion channels in which the effect of hydrophobic gating takes place.

## MATERIALS AND METHODS

The structure of open TRPV1 (PDB ID 7L2W, [4]) was inserted into a hydrated lipid bilayer with the following composition: 50% palmitoyloleoylphosphatidylcholine (POPC), 25% palmitoyloleoylphosphatidylethanolamine (POPE) and 25% cholesterol (about 900 molecules in the membrane). Na^+^ and Cl^–^ ions were added to ensure zero net charge at 0.15 M ionic concentration. MD simulations were carried out using GROMACS 2021.4 package [15], Amber99sd-ildn force field [16] and the TIP3P water model [17]. The modified Lennard-Jones parameters for Na^+^ and Cl^–^ developed by Joung and Cheatham [18] were used because of their better optimization for modeling of ion-water interactions. The actual values of σ and ε for Na^+^ were 2.439 Å and 0.3658 kJ/mol, for Cl^–^ – 4.478 Å and 0.1489 kJ/mol. Three independent 200 ns trajectories were calculated and analyzed.

The pore axis (*Z*) was defined as a line perpendicular to the membrane plane, *Z* = 0 was a center of mass of C_α_ atoms of residues 642, 643, 644, 645, 671, 675, 676, 679, 680, 683, 686, 687. Spatial distributions of water molecules, Na^+^ and hydrogen bonds were calculated as their densities averaged over the MD trajectory and shown as isosurfaces analogous to the work [3]. Two-dimensional density distributions were obtained from the spatial distributions. Radius of the coordination sphere of Na^+^ ions was taken to be 3.25 Å.
